# Acerola (*Malpighia emarginata* DC.) Juice Intake Suppresses UVB-Induced Skin Pigmentation in SMP30/GNL Knockout Hairless Mice

**DOI:** 10.1371/journal.pone.0170438

**Published:** 2017-01-23

**Authors:** Yasunori Sato, Eriko Uchida, Hitoshi Aoki, Takayuki Hanamura, Kenichi Nagamine, Hisanori Kato, Takeshi Koizumi, Akihito Ishigami

**Affiliations:** 1 Molecular Regulation of Aging, Tokyo Metropolitan Institute of Gerontology, Tokyo, Japan; 2 Corporate Science Research Division, Nichirei Corporation, Chiba, Japan; 3 Research and Development Division, Nichirei Foods Corporation, Chiba, Japan; 4 Research and Development Division, Research and Development Center, Nichirei Bioscience Corporation, Tokyo, Japan; 5 Corporate Sponsored Research Program ‘Food for Life’, The University of Tokyo, Tokyo, Japan; University of Alabama at Birmingham, UNITED STATES

## Abstract

**Background/Aims:**

Acerola (*Malpighia emarginata* DC.) is a fruit that is known to contain high amounts of ascorbic acid (AA) and various phytochemicals. We have previously reported that AA deficiency leads to ultraviolet B (UVB)-induced skin pigmentation in senescence marker protein 30 (SMP30)/gluconolactonase (GNL) knockout (KO) hairless mice. The present study was undertaken to investigate the effects of acerola juice (AJ) intake on the skin of UVB-irradiated SMP30/GNL KO mice.

**Research design/Principal findings:**

Five-week old hairless mice were given drinking water containing physiologically sufficient AA (1.5 g/L) [AA (+)], no AA [AA (-)] or 1.67% acerola juice [AJ]. All mice were exposed to UVB irradiation for 6 weeks. UVB irradiation was performed three times per week. The dorsal skin color and stratum corneum water content were measured every weekly, and finally, the AA contents of the skin was determined. The skin AA and stratum corneum water content was similar between the AA (+) and AJ groups. The L* value of the AA (+) group was significantly decreased by UVB irradiation, whereas AJ intake suppressed the decrease in the L* value throughout the experiment. Moreover, in the AJ group, there was a significant decrease in the expression level of dopachrome tautomerase, an enzyme that is involved in melanin biosynthesis.

**Conclusion:**

These results indicate that AJ intake is effective in suppressing UVB-induced skin pigmentation by inhibiting melanogenesis-related genes.

## Introduction

Senescence marker protein-30 (SMP30) was originally identified as a 34-kDa protein whose expression in the liver decreases with aging in an androgen-independent manner [1]. Previously, we found that SMP30 acts as a gluconolactonase (GNL) (EC 3.1.1.17) and is responsible for the conversion of L-gulonic acid into L-gulono-γ-lactone during the penultimate step of ascorbic acid (AA) synthesis [2]. Additionally, we demonstrated that SMP30/GNL knockout (KO) mice are unable to synthesize AA *in vivo* and develop scurvy when fed an AA-deficient diet [2]. Because humans cannot synthesize AA due to numerous mutations in the gene for L-gulono-γ-lactone oxidase, which is the terminal enzyme of the AA synthetic pathway, SMP30/GNL KO mice have been utilized to investigate the role of AA *in vivo* [3]. Thus far, we have reported that a complete lack of AA leads to an increase in superoxide generation in the brain [4, 5], an increase in protein oxidation in the liver [6, 7], the development of pulmonary emphysema [8–10], and decreases in the noradrenaline and adrenaline contents of the adrenal glands [11]. In this manner, we have revealed the physiological functions of AA by utilizing SMP30/GNL KO mice as an animal model. Furthermore, to clarify the pivotal role of AA in the skin, we developed and established SMP30/GNL KO hairless mice by mating SMP30/GNL KO mice with hairless mice [12]. The characteristic features of the SMP30/GNL KO hairless mice are as follows: an inability to synthesize AA, hairlessness, and the ability to undergo normal melanogenesis. We have reported that AA-deficient SMP30/GNL KO hairless mice with chronic UVB irradiation exhibit suppressed epidermal hyperplasia and develop excessive skin pigmentation [12]. Because SMP30/GNL KO hairless mice derive AA primarily through dietary intake, SMP30/GNL KO hairless mice can be valuable for evaluating not only AA but also the functional foods that promote AA in the skin.

Acerola (*Malpighia emarginata* DC.) is a fruit that is found throughout Central America and within the northern part of South America. Because acerola has high concentrations of AA in the edible part, it is considered to be one of the best sources of AA [13]. Additionally, acerola contains numerous functional phytochemicals, such as carotenoids and polyphenols [14–16]. Previously, we tested for various polyphenols in acerola and found that cyanidin-3-α-O-rhamnoside, pelargonidin-3-α-O-rhamnoside, quercetin-3-α-O-rhamnoside, kaempferol glycosides, astilbin, and proanthocyanidin are present [14, 15, 17]. In addition to polyphenols, we have reported on the contents of various nutritional components such as glucose, fructose, malic acid and several amino acids in the edible part of acerola [17]. The contents of glucose, fructose, malic acid, total polyphenols and total amino acids in acerola was 15 to 16, 14 to 16, 4 to 5, 1.2 to 1.5, and 1.8 to 2.1 g/kg edible portion, respectively [17]. It has been demonstrated that acerola intake exerts protective effects against DNA damage generated by FeSO_4_ [18], inhibits lung tumorigenesis induced by nicotine-derived nitrosamine ketone [19], regulates blood glucose levels and exerts anti-inflammatory effects in obese mice [20, 21]. Similarly, we have demonstrated a beneficial effect of acerola in humans; i.e., the intake of acerola juice (AJ) affects the absorption of AA in plasma and minimizes its excretion via urine in healthy Japanese subjects, which suggests improvement of the bioavailability of AA in humans [22]. In the skin, we have previously reported that the intake of acerola polyphenol extracts prevents UVB-induced skin pigmentation in brownish guinea pigs, which possess moderate numbers of melanocytes and melanosomes in the epidermis [23]. Because acerola contains not only a large amount of AA but also beneficial ingredients as noted above, it is important to elucidate the effect of whole juice intake in UVB-irradiated skin. Therefore, in this study, we focused on the effects of AJ intake on UVB-induced skin damage in SMP30/GNL KO hairless mice.

## Materials and Methods

### Preparation of acerola juice

AJ was prepared from concentrated mature clear juice, which was purchased from Nichirei do Brazil (Recife, Brazil). The concentrated clear juice was diluted with a hydrochloric acid solution and then filtered (No. 5C, Toyo Advantec Co., Tokyo, Japan). The prepared AJ was immediately frozen and stored at—80°C until use.

### Animals

The animal experiments were performed in accordance with the animal care and use protocol approved by the Institutional Animal Care and Use Committee of Tokyo Metropolitan Institute of Gerontology (TMIG) and in accordance with the Guidelines for the Care and Use of Laboratory Animals of TMIG.

SMP30/GNL KO hairless mice were established as previously described [12]. Briefly, SMP30/GNL KO hairless mice were generated by mating SMP30/GNL KO mice (C57BL/6 background) with hairless mice (Hos:HR-1, Hoshino Laboratory Animals, Ibaraki, Japan). A minimum of three generations of this strain were interbred for the present study. After weaning at 5 weeks old, male SMP30/GNL KO hairless mice were divided into the following three groups: AA deficient [AA (-)], AA supplemented [AA (+)], and AJ supplemented [AJ]. The AA (+) group had free access to water containing 1.5 g/L AA and 10 μM EDTA, whereas the AA (-) group was given water without AA. The AJ group was given 1.67% acerola juice diluted with the same drinking water that was given to the AA (-) group. The diluted AJ contained 1.5 g/L AA. The concentration of AA in the drinking water was sufficient to maintain normal AA levels in many tissues [24, 25]. Throughout the experiment, the drinking water was changed every 3 or 4 days. All mice were fed an AA-deficient diet (CL-2, CLEA Japan, Tokyo, Japan), and a 12-h light/dark cycle was maintained in a controlled environment.

### UVB irradiation of SMP30/GNL KO hairless mice

UVB irradiation was performed as previously described [12, 26]. SMP30/GNL KO hairless mice were irradiated with 94 mJ/cm^2^ of UVB three times per week for 6 weeks under three UVB lamps (GL20SE, Sankyo, Kanagawa, Japan). The irradiance of the UVB lamps was measured using a UV radiometer (UV-340, Custom, Tokyo, Japan). The UVB lamps emit a continuous light spectrum between 280–380 nm with a peak emission at 306 nm. At the end of the six-week experimental period, the mice were euthanized by cervical dislocation, and their skin was subsequently collected and stored at -80°C until use.

### Measurements of dorsal skin color and stratum corneum water content

During the experiment, the dorsal skin color and stratum corneum water content were measured every week for 6 weeks. The degree of dorsal skin color was quantified using a portable reflectance colorimeter in the CIE-L*, a*, b* system (Minolta CR-200 colorimeter, Konica Minolta, Tokyo, Japan) by measuring the reflected object color. The results are expressed as an L* value, which is a measurement of skin lightness on a continuous black to white scale, where 100 is completely white and 0 is completely black. The stratum corneum water content, as assessed indirectly by high-frequency superficial skin conductance, was measured using a Skicon-200 (IBS Co., Hamamatsu, Japan).

### Measurement of AA and DHA contents in the skin

The skin levels of AA and dehydroascorbic acid (DHA), which is an oxidized form of AA, were measured using HPLC and electrochemical detection (ECD), as previously reported [12, 27]. Briefly, the skin was homogenized with 14 volumes of ice-cold 5.4% metaphosphoric acid (MPA) containing 1 mM EDTA (MPA/EDTA) solution and centrifuged at 21,000 x g for 10 min at 4°C. The total AA (AA + DHA) and AA contents in the centrifugal supernatants were determined individually as follows, and the DHA content was calculated by subtracting the AA content from the total AA content. To determine of the total AA, the centrifugal supernatants were reduced with Tris(2-carboxyethyl)phosphine hydrochloride (TCEP) for 2 h on ice. After the reduction, the reaction mixture was diluted with 5% MPA/EDTA and analyzed for total AA by HPLC coupled with ECD. For the determination of AA, the non-reduced centrifugal supernatants were diluted with 5% MPA/EDTA and analyzed by HPLC coupled with ECD. Separation was achieved on an Atlantis dC18 5-μm column (4.6 × 150 mm) combined with an Atlantis dC18 5-μm guard column (4.6 × 20 mm) from Nihon Waters (Tokyo, Japan). The mobile phase consisted of 50 mM phosphate buffer (pH 2.8), 540 μM EDTA and 2% methanol. The flow rate was 1.3 mL/min. The temperatures for the column and Waters 2465 electrochemical detector were set at 30°C, and the temperature for the autosampler thermostat was set at 4°C. The sample was injected into the HPLC coupled with ECD for the AA analysis. Electrical signals were recorded using an electrochemical detector with a glassy carbon electrode at +0.6 V. All electrical signal data from the electrochemical detector were collected using Waters Empower2 software (Nihon Waters, Tokyo, Japan).

### Histology

Dorsal skin tissues were fixed in a 10% formalin neutral buffer solution (Wako Pure Chemicals, Osaka, Japan), embedded in paraffin, and sectioned on a microtome at a thickness of 3 μm. For the hematoxylin and eosin (HE) staining, the sections were deparaffinized with xylene, rehydrated with a graded series of ethanol, and then subjected to HE staining. The epidermal thicknesses were determined using the Multi Gauge, version 3.0 (Fuji Photo Film, Tokyo, Japan).

### Quantitative real-time RT-PCR analysis

Total RNA was extracted from the skin tissues using TRIzol reagent (Invitrogen, Karlsruhe, Germany) according to the manufacturer’s protocol. The total RNA (1 μg) was reverse transcribed at 37°C using PrimeScript^™^ RT Enzyme (Takara, Shiga, Japan). Real-time RT-PCR was performed using a real-time PCR detection system (Takara Bio, Madison, WI, USA), and expression of the following genes was detected in the AA (-), AA (+), and AJ groups: tyrosinase (*Tyr*), tyrosinase related protein-1 (*Tyrp1*), dopachrome tautomerase (*Dct*), *Tnf-α*, endothelin 1 (*Edn1*), and cyclin D1 (*Ccnd1*). The final mixture for RT-PCR consisted of 1× SYBR Premix Ex Taq Mix (Takara), 0.4 μM of primers (forward and reverse), and cDNA. The primer sequences are provided in Table 1. The PCR amplification consisted of 40 cycles (95°C for 5 s and 60°C for 30 s) after an initial denaturation step (95°C for 10 s). The mRNA expression levels were evaluated relative to the level of ribosomal protein, large, P1 (*Rplp1*), and the mRNA levels of the AA (+) group were designated as 1.0.

**Table 1:** Primer sets used for qPCR analysis.

Target gene		Sequence
*Tyrosinase (Tyr)*	Forward	5'-CAAGTACAGGGATCGGCCAAC-3'
	Reverse	5'-GGTGCATTGGCTTCTGGGTAA-3'
*Tyrosinase-related protein 1 (Tyrp1)*	Forward	5'-TGATGCGGTCTTTGACGAATG-3'
	Reverse	5'-GTTGGTAACTGGAGGCCAGAATG-3'
*Dopachrome tautomerase (Dct)*	Forward	5'-AACCGCAGAGCAACTTGGCTAC-3'
	Reverse	5'-CTCCCAGGATTCCAATGACCAC-3'
*Tnf-α*	Forward	5'-TTGTTGCCTCCTCTTTTGCT-3'
	Reverse	5'-TGGTCACCAAATCAGCGTTA-3'
*Endothelin 1 (Edn1)*	Forward	5'-GTGTTCCCTAGCCTGTCTGC-3'
	Reverse	5'-TGGAATCTCCTGGCTCTCTG-3'
*Cyclin D (Ccnd1)*	Forward	5'-ATTTGCACACCTCTGGCTCT-3'
	Reverse	5'-TCACCTCTTCCCTCACATCC-3'
*Ribosomal protein*, *large*, *P1 (Rplp1)*	Forward	5’-TCCGAGCTCGCTTGCATCTA-3’
	Reverse	5’-CAGATGAGGCTCCCAATGTTGA-3’

### Statistical analysis

The probability of significant differences between the experimental groups was determined by one-way ANOVA followed by Tukey’s HSD post hoc comparisons. Dunnett's test was also used to assess the effects of UVB on the L* value. KaleidaGraph software was used (Synergy Software, Reading, PA, USA) to perform the one-way ANOVAs. Differences were considered significant at *P* values of <0.05.

## Results

### AA concentration in acerola juice

To adjust the AA concentration in the drinking water to 1.5 g/L, we first determined the total AA concentration in the AJ. As illustrated in Table 2, the total AA, AA, and DHA concentrations were 89.5 ± 1.5, 85.9 ± 2.2, and 3.6 ± 0.7 g/L, respectively. Because DHA can be reduced to AA by several enzymes, including glutaredoxin, protein disulfide isomerase, omega class glutathione transferase, 3α-hydroxysteroid dehydrogenase, and thioredoxin reductase [28], we used the total AA concentration in the AJ for the preparation of the drinking water for the AJ group. That is, the AJ group was given 1.67% AJ, which contained 1.5 g/L total AA.

**Table 2:** Total AA, AA, and DHA concentrations in acerola juice.

	Total AA (g/L)	AA (g/L)	DHA (g/L)
Acerola juice	89.5 ± 1.5	85.9 ± 2.2	3.6 ± 0.7

The data are presented as the mean ± SEM of three acerola juice samples.

### Body weight in skin

To investigate the influence of AJ intake on the growth of the SMP30/GNL KO hairless mice, we measured the body weight changes of the three groups during the experiment. The body weights of the mice did not vary significantly among the three groups from the start date to the fifth week of the experimental period, but the body weight of the AA (-) group was significantly lower than that of the AJ group at 6 weeks (Table 3).

**Table 3:** Body weight change.

	AA (-) (g)	AA (+) (g)	AJ (g)
Start date	16.7 ± 0.8	16.7 ± 0.8	16.5 ± 1.2
1 w	18.2 ± 1.3	16.3 ± 0.7	18.0 ± 0.8
2 w	20.1 ± 0.9	18.0 ± 0.9	18.8 ± 0.7
3 w	21.3 ± 0.8	19.4 ± 0.9	19.9 ± 0.7
4 w	21.7 ± 0.8	20.7 ± 0.9	21.0 ± 0.5
5 w	19.2 ± 0.9	20.3 ± 1.1	20.2 ± 0.6
6 w	18.6 ± 1.0	21.3 ± 0.9	22.0 ± 0.6*

The values are expressed as the means ± the SEMs of five animals. ANOVA analysis F_2, 12_ = 4.6, *P* < 0.05 for 6 weeks. Asterisk indicate significant comparisons (*P < 0.05) between the AA (-) and AJ groups by Tukey’s HSD post hoc comparisons.

### AA content in skin

**Fig 1:**
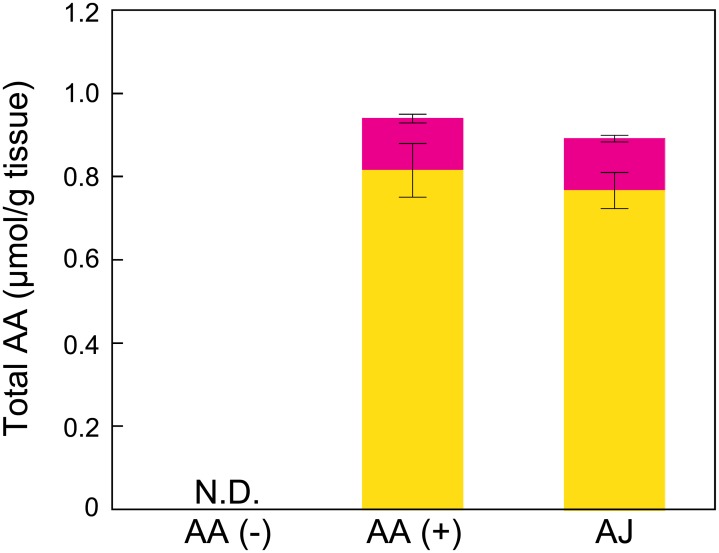
AA and DHA contents. The AA content (yellow bar) was determined by HPLC coupled with ECD, as described in the Materials and Methods section. The total AA (total bar height) content was determined by the reduction of DHA with TCEP. The DHA content (red bar) was calculated by subtracting the AA content from the total AA content determined in a different chromatographic run. N.D. indicates not detected. The values are expressed as the means ± the SEMs of five animals. ANOVA analysis: F_2, 12_ = 104.3, *P* < 0.001 for the AA content and F_2, 12_ = 90.9, *P* < 0.001 for the DHA content. The error bars represent the SEMs for the DHA and AA. AA, ascorbic acid; AJ, acerola juice.

At the end of the experiment (6 weeks), the AA and DHA content in the skin of the AA (-) group was below the level of detection (Fig 1). In contrast, the AA and DHA content was not significantly different between the AA (+) and AJ groups. The amounts of water consumption in the AA (-), AA (+), and AJ groups were 4.9 ± 0.4, 4.1 ± 0.3, and 4.7 ± 0.3 mL/day/mouse, respectively. The consumption of total AA in the AA (+) and AJ groups were 6.2 ± 0.5, and 7.1 ± 0.5 mg/day/mouse, respectively. There was no significant difference in the amount of water ingested during the experiment. Moreover, there was no significant difference in the consumption of total AA between the AA (+) and AJ groups.

### Histological skin analysis

**Fig 2:**
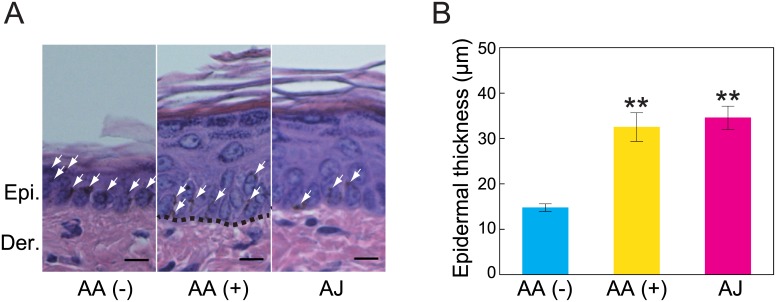
The effect of acerola juice intake on epidermal morphology and thickness. (A) High magnifications of the epidermis from the skins of the AA (-), AA (+) and AJ groups are shown. The white arrows indicate the skin pigment. (B) The epidermal thicknesses in the HE-stained sections were measured as the distance between the top of the basement membrane and the bottom of the stratum corneum in five fields randomly selected from each animal. The values are expressed as the means ± the SEMs of five animals. ANOVA analysis: F_2, 12_ = 20.6, *P* < 0.001. The asterisk indicates a significant difference (**P < 0.01) compared with the AA (-) group by Tukey’s HSD post hoc comparisons. AA, ascorbic acid; AJ, acerola juice; Der., dermis; Epi., epidermis. Bar = 10 μm.

To investigate the effects of AJ intake on the histologically evaluated changes in the skin, we performed HE staining and measured the thicknesses of the epidermal skins of all the mice. Representative histological HE skin staining is provided in Fig 2A. In normal skin, chronic UVB irradiation induces epidermal hyperplasia, which is important for protecting the basal keratinocytes from subsequent DNA damage from UVB [29, 30]. As expected, epidermal hyperplasia was observed in the AA (+) and AJ groups (Fig 2A). The epidermal thicknesses were not significantly different between the AA (+) and AJ groups (Fig 2B). However, the AA (-) group did not manifest epidermal hyperplasia. Massive skin pigmentation was observed in the epidermis in the AA (-) group, but only slight pigmentation was observed in the AA (+) and AJ groups (Fig 2A). The epidermal thickness in the AA (-) group was significantly decreased compared with those of the other two groups (Fig 2B).

### Stratum corneum water content

**Fig 3:**
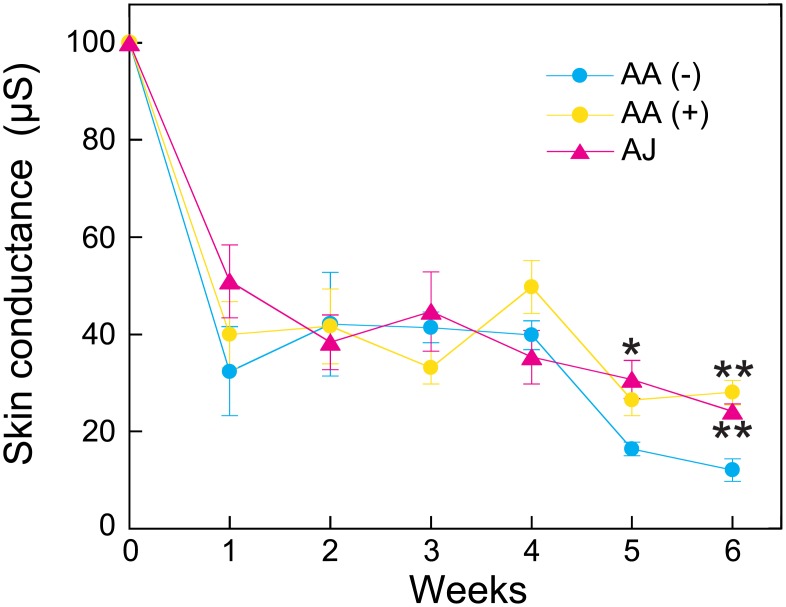
The effect of acerola juice intake on the stratum corneum water content. The stratum corneum water content was measured every week for 6 weeks as described in the Materials and Methods. The values are expressed as the means ± the SEMs of five animals. ANOVA analysis: F_2, 12_ = 4.6, *P* < 0.05 at 5 weeks and F_2, 12_ = 25.2, *P* < 0.001 at 6 weeks. The asterisks indicate significant differences (*P < 0.05, **P < 0.01) compared with the AA (-) group by Tukey’s HSD post hoc comparisons. AA, ascorbic acid; AJ, acerola juice.

Because chronic UVB irradiation has been known to cause skin barrier dysfunction as well as decreases in the water content of the stratum corneum [31], we measured the water content of the stratum cornea of the three groups during the experiment. As illustrated in Fig 3, the stratum corneum water content decreased gradually in all groups after the initiation of UVB irradiation. The stratum corneum water content was not significantly different between the AA (+) and AJ groups during the experiment. However, the water content of the AJ group was significantly higher than that of the AA (-) group at 5 and 6 weeks. Moreover, the water content of the AA (+) group was significantly higher than that of the AA (-) group only at 6 weeks.

### UVB-induced skin pigmentation

**Fig 4:**
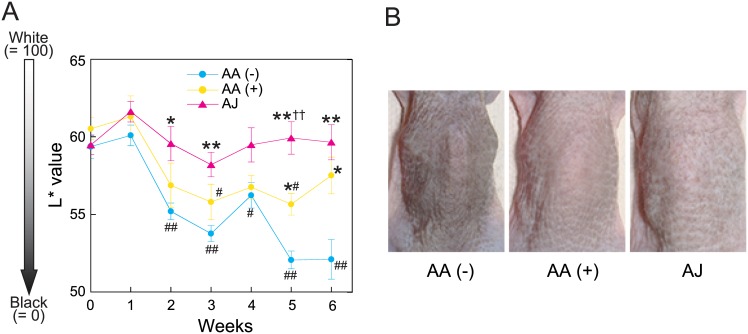
The effects of acerola juice intake on L* value and back skin colors. (A) The time course of the L* value (a measure of skin lightness). (B) Representative photographs of UVB-irradiated areas at 6 weeks. The values are expressed as the means ± the SEMs of five animals. ANOVA analysis: F_6, 28_ = 17.4, *P* < 0.0001 for the AA (-) group, F_6, 28_ = 4.4, *P* < 0.01 for the AA (+) group, and F_6, 28_ = 1.1, *P* = 0.39 for the AJ group. Sharps indicate significant differences (^#^*P* < 0.05, ^##^*P* < 0.01) compared with the start date by Dunnett's test for post hoc comparisons. ANOVA analysis: F_2, 12_ = 4.1, *P* < 0.05 at 2 weeks, F_2, 12_ = 6.7, *P* < 0.01 at 3 weeks, F_2, 12_ = 3.8, *P* = 0.053 at 4 weeks, F_2, 12_ = 24.2, *P* < 0.0001 at 5 weeks, and F_2, 12_ = 10.5, *P* < 0.01 at 6 weeks. The asterisks indicate significant differences (*P < 0.05, **P < 0.01) compared with the AA (-) group by Tukey’s HSD post hoc comparisons. The double daggers indicate significant differences (^††^*P* < 0.01) compared with the AA (+) group by Tukey’s HSD post hoc comparisons. AA, ascorbic acid; AJ, acerola juice.

We next assessed the effects of AJ intake on the UVB-induced skin pigmentation of the SMP30/GNL KO hairless mice every week using a colorimeter. The L* values decreased gradually in the AA (-) and AA (+) group after the initiation of UVB irradiation (Fig 4A). Compared with the start day in the same group, significant differences were observed after 2 weeks of irradiation in the AA (-) group, whereas such differences were only observed at 3 and 5 weeks in the AA (+) group. Surprisingly, the L* value of the AJ group did not change significantly during the experiment compared with the value on the start day. The L* value of the AJ group was significantly higher than that of the AA (-) group after 2 weeks of irradiation, except at week 4. In contrast, the L* value of AA (+) group was significantly higher than that of the AA (-) group at weeks 5 and 6. Comparison between the AJ and AA (+) groups revealed that the L* value of the AJ group was higher throughout the experiment and significantly higher than that of the AA (+) group at 5 weeks. Fig 4B provides representative photographs of the dorsal skin colors at the end of the experiments. There was a visible reduction in the pigmentation of the skin that had been treated with AJ.

### Gene expression

**Fig 5:**
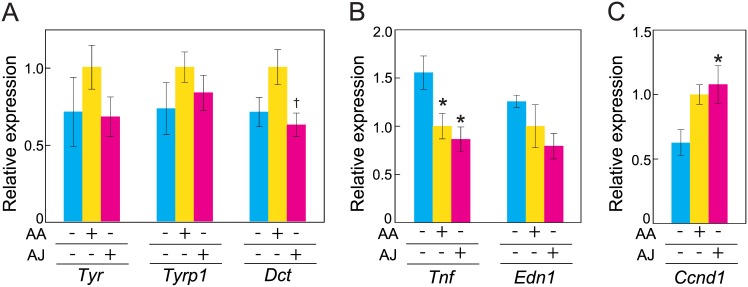
The effect of acerola juice intake on gene expression in the skin. (A) Melanogenesis-related enzyme genes *Tyr*, *Tyrp1* and *Dct*. (B) Cytokine genes *Tnf-a* and *Edn1*. (C) Cell cycle progression gene *Ccnd1*. Quantifications of the mRNA are illustrated relative to the *Rplp1* mRNA. The mRNA levels of the AA (+) group were designated as 1.0. The values are expressed as the means ± the SEMs of five animals. ANOVA analysis: F_2, 12_ = 1.1, *P* = 0.369 for the *Tyr* gene, F_2, 12_ = 1.1, *P* = 0.372 for the *Tyrp1* gene, F_2, 12_ = 4.1, *P* < *0*.*05* for the *Dct* gene, F_2, 12_ = 6.3, *P* < *0*.*05* for the *Tnf-a* gene, F_2, 12_ = 2.3, *P* = 0.147 for the *Edn1* gene, and F_2, 12_ = 4.7, *P* < *0*.*05* for the *Ccnd1* gene. The asterisks indicate significant differences (*P < 0.05) compared with the AA (-) group by ANOVA followed by Tukey HSD post hoc comparisons. The daggers indicate significant differences (^†^*P* < 0.05) compared with the AA (+) group by ANOVA followed by Tukey’s HSD post hoc comparisons. AA, ascorbic acid; AJ, acerola juice.

To determine the effects of AJ intake on gene expression, we next examined the mRNA expression levels of melanogenesis-related genes (*Tyr*, *Tyrp1*, and *Dct*), inflammatory cytokines (*Tnf-α* and *Edn1*) and a cell cycle-related gene (*Ccnd1*) in the skins of all groups. The *Tyr* mRNA expression levels of the AA (-) and AJ groups were 29% and 32% lower, respectively, than that of the AA (+) group, although these differences were not statistically significant (Fig 5A). Similarly, the *Tyrp1* mRNA expression levels of the AA (-) and AJ group were 27% and 17% lower, respectively, than that of the AA (+) group, although these differences were not statistically significant (Fig 5A). The *Dct* mRNA expression levels of the AA (-) and AJ groups were 29% and 37% lower, respectively. Of note, there was a significant difference in the *Dct* mRNA expression levels between the AA (+) and AJ groups (Fig 5A). As illustrated in Fig 5B, the *Tnf-a* mRNA expression level of the AA (-) group was significantly higher than those of the AA (+) and AJ groups. In contrast, there was no significant difference in the level of *Tnf-α* mRNA between the AA (+) and AJ groups. The *Edn1* mRNA expression level of the AA (-) group was 26% higher than that of the AA (+) group (Fig 5B). Although there was no significant difference between the AA (+) and AJ groups, the *Edn1* mRNA expression level of the AJ group was 21% lower than that of the AA (+) group. Compared with the AA (+) group, the *Ccnd1* mRNA expression level of the AA (-) group was relatively lower by 37%, although this difference did not reach significance (*p* = 0.08; Fig 5C). There was no significant difference between the AA (+) and AJ groups in the *Ccnd1* mRNA levels. However, the *Ccnd1* mRNA expression level of the AJ group was significantly higher than that that of the AA (-) group.

## Discussion

Acerola is considered a functional fruit due to its strong antioxidant properties and high phenolic contents and is thus consumed worldwide to promote health [14]. Previously, acerola has been demonstrated to exhibit beneficial effects, such as anti-mutagenic activity, an anti-hyperglycemic effect, and an *in vivo* anti-inflammatory effect [18–21]. Because little is known about the protective effects of AJ intake in UVB-damaged skin, we examined the effects of AJ intake on the skin of SMP30/GNL KO hairless mice. In the present study, we demonstrated that AJ intake suppresses excessive UVB-induced skin pigmentation compared with the same amount of AA intake.

In mammalian species, two main types of melanin are generated, that is, a red-to-yellow pheomelanin and a brown-to-black eumelanin [30]. It has been appreciated that melanin is the major pigment of the skin and determines the skin color. Skin pigmentation is the major photo-protective response of the skin against chronic UV radiation [32]. To elucidate the mechanism of the suppressive effect of AJ intake on skin pigmentation, we investigated the effect of AJ intake on the expression of melanogenesis-related genes. Tyrosinase is an essential enzyme in melanogenesis and catalyzes L-tyrosine to L-DOPA and L-DOPA to dopaquinone. Dopachrome tautomerase catalyzes dopachrome to dihydroxyindole-2-carboxylic acid (DHICA). Tyrosinase-related protein-1 is a DHICA oxidase that catalyzes DHICA to eumelanin [32]. We found that AJ intake significantly decreased the expression level of *Dct* and tended to decrease the levels of *Tyr* and *Tyrp1*. We previously investigated variations in the polyphenol composition of acerola and found that cyanidin-3-α-O-rhamnoside, pelargonidin-3-α-O-rhamnoside, quercetin-3-α-O-rhamnoside, kaempferol glycosides, astilbin and proanthocyanidin are present [14, 15, 17]. Additionally, we revealed that polyphenol extracts from acerola and individual polyphenols (cyanidin-3-α-O-rhamnoside, pelargonidin-3-α-O-rhamnoside and Astilbin) inhibit tyrosinase activity [23]. Previous studies have indicated that polyphenol extracts inhibit melanogenesis through the down-regulations of *Tyr*, *Tyrp1*, and *Dct* [33–35]. Therefore, the suppressive effect of AJ intake on skin pigmentation may have been due to the inhibitory effects of polyphenols on melanogenesis.

Moreover, UVB exposure facilitates the release of pro-inflammatory mediators, including TNF-α and Edn1, from keratinocytes [36–38]. TNF-α stimulates the infiltration of immune cells into the skin, and elastases and collagenases are thereby secreted from the infiltrated immune cells, leading to damage in the skin [39]. Considering that there was no difference in *Tnf-a* mRNA expression between the AJ and AA (+) groups, it appears that AJ can suppress the inflammation induced by UVB. Edn1 is considered one of the most potent agonists for stimulating melanocytes to accelerate melanogenesis [40, 41]. Because the *Edn1* mRNA level in the AJ group was lower than that in the AA (+) group, AJ intake may suppress melanogenesis-related cytokines.

Because keratinocytes differentiate and migrate to the upper layers of the epidermis, melanin pigment is removed from the epidermis along with keratinocytes. We found that the *Ccnd1* mRNA expression level of the AJ group was significantly higher than that of the AA (-) group. Cyclin D1 is a critical gene for the progression from the G_0_/G_1_ to the S phase of the cell cycle and thus contributes to keratinocyte differentiation [42]. Robles *et al*. reported that the overexpression of cyclin D1 increases the rate of epidermal proliferation in cyclin D1 transgenic mice [43]. Our results revealed that AJ intake may facilitate the removal of melanized keratinocytes from the epidermis.

Another possibility is the involvement of effects of reactive oxygen species (ROS) on melanogenesis in the skin. UV interacts with oxygen to promote the formation of ROS, such as superoxide radicals, hydrogen peroxide, and highly reactive hydroxyl radicals in the skin [30]. It has been reported that the UV radiation-induced proliferation and melanogenesis of melanocytes are reduced by the topical application of an antioxidant, such as AA or vitamin E, to the skin of hairless mice, which suggests the involvement of ROS in melanogenesis in melanocytes [44]. Mitra *et al*. also reported that oxidative stress appears to have a role in pheomelanin-mediated melanogenesis [45]. Our previous study demonstrated that polyphenols isolated from the acerola fruit exert superoxide radical scavenging activity using an electron spin resonance spectrometer [14]. Other studies have also indicated that acerola has antioxidant activity using a 1, 1-diphenyl-2-picrylhydrazyl radical scavenging assay [16, 46, 47]. Some studies have reported that the antioxidant activity of acerola juice depends on its polyphenols and AA contents [21, 48]. Because there was no difference in AA content between the AA (+) and AJ groups, as illustrated in Fig 1, various polyphenols from acerola juice may contribute to the reduction of UVB-induced skin pigmentation.

In addition, it has been reported that cortisol and some neuroendocrine mediators such as melatonin and β-endorphin are involved in melanogenesis and UVB-induced skin damage. Cortisol can be produced in the skin by UVB irradiation [49]. It is suggested that production of cortisol maintains skin homeostasis against UVB-induced skin damage [49]. Melatonin is mainly produced in the pineal gland and retina, however, it is also produced in the skin [50–53]. Janjetovic *et al*. reported that melatonin has strong radical-scavenging activities and has a protective effect against UBV-induced oxidative stress [53]. Moreover β-endorphin can be detected in the components of the skin such as the epidermis, dermis and adnexa [50, 54]. It has been reported that β-endorphin stimulates keratinocyte migration in vitro, induces epidermal and follicular melanogenesis. Additionally, melanocortin receptor 1 is recognized for its role in the regulation of melanin pigmentation [55]. Although the relationship between acerola and these molecules are unclear, acerola may coordinate with these molecules to protect against UVB-induced damage.

We previously reported that AA deficiency suppresses UVB-induced epidermal hyperplasia and increases UVB-induced skin pigmentation [12]. In this study, we confirmed the same results, as illustrated in Figs 2 and 4. Moreover, we found for the first time that AA deficiency decreased the stratum corneum water content and significantly increased the expression level of *Tnf-a*. These results suggest that AA deficiency facilitates the release of pro-inflammatory mediators induced by UVB radiation and leads to epidermal barrier dysfunction. Moreover, our study also indicates that SMP30/GNL KO hairless mice represent an effective animal model for investigating the effects of functional foods on the skin.

In conclusion, our findings in this study strongly indicate that the intake of AJ compared with AA intake in the same amount significantly prevented skin damage induced by long-term UVB irradiation. Our findings strongly suggest that acerola is not only one of the best sources of AA but is also a functional food for the skin. Further study is needed to elucidate the protective effects on human skin in a human trial or using a reconstructed human skin model.

## Abbreviations

AA: ascorbic acid
AJ: acerola juice
Ccnd1: cyclin D1
Dct: dopachrome tautomerase
DHA: dehydroascorbic acid
DHICA: dihydroxyindole-2-carboxylic acid
ECD: electrochemical detection
Edn1: endothelin 1
GNL: gluconolactonase
KO: knockout
MPA: metaphosphoric acid
SMP30: senescence marker protein 30
TCEP: tris(2-carboxyethyl)phosphine hydrochloride
Tyr: tyrosinase
Tyrp1: tyrosinase related protein-1
UVB: ultraviolet B
